# Supplementary material for: The components of an electrical synapse as revealed by expansion microscopy of a single synaptic contact
Source: eLife. 2024 Jul 12;13:e91931. doi: 10.7554/eLife.91931 (PMC11333041; doi:10.7554/eLife.91931)
Supplement: Supplementary file 1. [file elife-91931-supp1.pdf]

**Key Resources Table**				
**Reagent type (species) or resource**	**Designation**	**Source or reference**	**Identifiers**	**Additional information**
strain, strain background (*Danio rerio*)	Tol-056 enhancer trap line	PMID:19474306	ZFIN:ZDB-ALT-110217–6; PMID:19474306	Satou et al., 2009
antibody	mouse IgG1 anti-Cx35/36 (monoclonal)	Millipore	Millipore:MAB3045; RRID:AB_94632	(1:250)
antibody	rabbit anti-Cx35.5 (monoclonal)	PMID:25484298; Fred Hutch Antibody Technology Facility	FHATF:clone12H5	(1:200); Miller et al., 2015
antibody	mouse IgG2A anti-Cx34.1 (monoclonal)	PMID:25484298; Fred Hutch Antibody Technology Facility	FHATF:clone5C10A	(1:200); Miller et al., 2015
antibody	mouse IgG1 anti-ZO1 (monoclonal)	Invitrogen	Invitrogen:33-9100; RRID:AB_2533147	(1:200)
antibody	mouse IgG1 anti-N-cadherin (monoclonal)	BD Transduction Laboratories	BD:610920; RRID:AB_2077527	(1:50)
antibody	mouse IgG1 anti-Beta-catenin (monoclonal)	Sigma	Sigma:C7207; RRID:AB_476865	(1:100)
antibody	chicken IgY anti-GFP (polyclonal)	Abcam	Abcam:ab13970; RRID:AB_300798	(1:200)
antibody	rabbit IgG anti-GluR2/3 (polyclonal)	Millipore	Millipore:07-598; RRID:AB_11213931	(1:200)
antibody	mouse IgG- Alexa Fluor 546 (polyclonap)	Invitrogen	Invitrogen:A11030	(1:200)
antibody	mouse IgG- Alexa fluor 647 (polyclonal)	Invitrogen	Invitrogen:A21235	(1:200)
antibody	mouse IgG- Atto 647N (polyclonal)	Sigma-Aldrich	Sigma-Aldrich:50185	(1:200)
antibody	rabbit IgG– Alexa fluor 546 (polyclonal)	Invitrogen	Invitrogen:A11010	(1:200)
antibody	rabbit IgG- Atto 647N (polyclonal)	Sigma-Aldrich	Sigma-Aldrich:40839	(1:200)
antibody	chicken IgY- Alexa Fluor 488 (polyclonal)	Invitrogen	Invitrogen:A11039	(1:200)

chemical compound, drug	Acryloyl-X SE	Invitrogen	Invitrogen:A20770	
chemical compound, drug	1M Tris (pH 8.0)	Invitrogen	Invitrogen:AM9855G	
chemical compound, drug	0.5M EDTA	Invitrogen	Invitrogen:AM9260G	
chemical compound, drug	ddH2O (cell culture grade water)	Sigma-Aldrich	Sigma-Aldrich:W3500	
chemical compound, drug	N,N' methylenebisacrylamide	Sigma-Aldrich	Sigma-Aldrich:M7279	
chemical compound, drug	Ammonium Persulfate (APS)	Sigma-Aldrich	Sigma-Aldrich:A3678	
chemical compound, drug	4-hydroxy-TEMPO (4-HT)	Sigma-Aldrich	Sigma-Aldrich:176141	
chemical compound, drug	Sodium Acrylate	Sigma-Aldrich	Sigma-Aldrich:408220	
chemical compound, drug	acrylamide	Sigma-Aldrich	Sigma-Aldrich:A9099	
chemical compound, drug	Tetramethylethylenediamine (TEMED)	Sigma-Aldrich	Sigma-Aldrich:T7024	
chemical compound, drug	DMSO (Dimethyl sulfoxide)	Honeywell	Honeywell:67685	
chemical compound, drug	NaCl (Sodium Chloride)	Sigma-Aldrich	Sigma-Aldrich:S9888	
chemical compound, drug	10X PBS	Sigma-Aldrich	Sigma-Aldrich:6506-OP	
chemical compound, drug	100-X Triton	Sigma-Aldrich	Sigma-Aldrich:X100	
chemical compound, drug	MS-222 (tricaine methanesulphonate)	Sigma-Aldrich	Sigma-Aldrich:A5040	
chemical compound, drug	trichloroacetic acid	Sigma-Aldrich	Sigma-Aldrich:T6399	
chemical compound, drug	Normal Goat Serum	Vector Laboratories	Vector:S-1000	
chemical compound, drug	ProLong Gold antifade	Invitrogen	Invitrogen:P36930	
chemical compound, drug	Proteinase K	Thermo Fisher Scientific	TFS:EO0491	
chemical compound, drug	Poly-D-Lysine	Sigma-Aldrich	Sigma-Aldrich:A-003-E	
software, algorithm	GraphPad Prism	Graph Pad Software	https://www.graphpad.com/	
software, algorithm	Photoshop	Adobe	https://www.adobe.com/	Adobe Photoshop CC 2015
software, algorithm	Illustrator	Adobe	https://www.adobe.com/	Adobe Illustrator CC 2015
software, algorithm	FIJI	PMID:22743772	PMID:22743772; https://fiji.sc/	Schindelin et al., 2012

software, algorithm	ZEN (black edition)	Carl Zeiss Microscopy	https://www.micro-shop.zeiss.com/en/us/	
software, algorithm	Canvas	Canvas	https://www.canvasgfx.com/products/canvas-x-draw	
other	LSM 710 and LSM 880 Zeiss microscopes	Carl Zeiss Microscopy	https://www.zeiss.com/corporate/int/home.html	
other	40x 1.0 NA water immersion objective	Carl Zeiss Microscopy	CZM:421462-9900	
other	63x 1.40 NA oil immersion objective	Carl Zeiss Microscopy	CZM:420782-9900	
